# Arts, Narrative, and Creativity: Enhancing Connection, Communication, and Health

**DOI:** 10.1093/geroni/igaa057.2344

**Published:** 2020-12-16

**Authors:** Jacqueline Eaton

## Abstract

Older adults will comprise17% of the world’s population by2050. Meeting the unique challenges of an aging society requires innovative solutions to enhance the aging experience, reduce the pressures placed on the current infrastructure, and inspire future generations to see that aging matters. Shifts in multi and transdisciplinary approaches to research in aging have contributed to an increasing surge in the development and evaluation of arts-based strategies to find innovative solutions to the challenges associated with aging. This symposium examines the use of creative expression as a tool to enhance older adult health and quality of life through five different approaches targeting1) workforce training,2) mental health self-support,3) perceptions of peer loss and death in long-term care and,4) social activity for persons with dementia. Cox and Maguire enhance undergraduate gerontology courses with guided autobiography, portfolios, and creative collaging processes. Eaton et al. report findings from a study testing the use of arts-based caregiving techniques by certified nursing assistants to improve resident care. Fortuna and Brooks describe the use of narrative and storytelling to share lived experiences of aging with a mental health condition while promoting age-related self-management skills development. Olson presents findings from her work using expressive arts to explore perceptions of death and peer loss by residents in long-term care. Finally, Mittelman and Harris share outcomes from the program, A Place For Us, which provides creative expression opportunities for persons with dementia.

